# N-acetyl cysteine-loaded graphene oxide-collagen hybrid membrane for scarless wound healing

**DOI:** 10.7150/thno.34480

**Published:** 2019-08-12

**Authors:** Jialun Li, Chuchao Zhou, Chao Luo, Bei Qian, Shaokai Liu, Yuyang Zeng, Jinfei Hou, Bin Deng, Yang Sun, Jie Yang, Quan Yuan, Aimei Zhong, Jiecong Wang, Jiaming Sun, Zhenxing Wang

**Affiliations:** 1 Department of Plastic Surgery, Union Hospital, Tongji Medical College, Huazhong University of Science and Technology, Wuhan 430022, China; 2 Department of Pharmacy, Union Hospital, Tongji Medical College, Huazhong University of Science and Technology, Wuhan,430022, China; 3 Department of Medical Records Management and Statistics, Union Hospital, Tongji Medical College, Huazhong University of Science and Technology, Wuhan 430022, China

**Keywords:** collagen, graphene oxide, reactive oxygen species, NAC, wound healing

## Abstract

Wound dressings composed of natural polymers, such as type I collagen, possess good biocompatibility, water holding capacity, air permeability, and degradability, and can be used in wound repair. However, due to the persistent oxidative stress in the wound area, the migration and proliferation of fibroblasts might be suppressed, leading to poor healing. Thus, collagen-containing scaffolds are not suitable for accelerated wound healing. Antioxidant N-acetyl cysteine (NAC) is known to reduce the reactive oxygen species (ROS) and has been widely used in the clinic. Theoretically, the carboxyl group of NAC allows loading of graphene oxide (GO) for sustained release and may also enhance the mechanical properties of the collagen scaffold, making it a better wound-dressing material. Herein, we demonstrated an innovative approach for a potential skin-regenerating hybrid membrane using GO incorporated with collagen I and NAC (N-Col-GO) capable of continuously releasing antioxidant NAC.

**Methods:** The mechanical stability, water holding capacity, and biocompatibility of the N-Col-GO hybrid membrane were measured *in vitro*. A 20 mm rat full-skin defect model was created to evaluate the repair efficiency of the N-Col-GO hybrid membrane. The vascularization and scar-related genes in the wound area were also examined.

**Results:** Compared to the Col only scaffold, N-Col-GO hybrid membrane exhibited a better mechanical property, stronger water retention capacity, and slower NAC release ability, which likely promote fibroblast migration and proliferation. Treatment with the N-Col-GO hybrid membrane in the rat wound model showed complete healing 14 days after application which was 22% faster than the control group. HE and Masson staining confirmed faster collagen deposition and better epithelization, while CD31 staining revealed a noticeable increase of vascularization. Furthermore, Rt-PCR demonstrated decreased mRNA expression of profibrotic and overexpression of anti-fibrotic factors indicative of the anti-scar effect.

**Conclusion:** These findings suggest that N-Col-GO drug release hybrid membrane serves as a better platform for scarless skin regeneration.

## Introduction

Skin injuries resulting from surgery, trauma, diabetes, or chronic wounds, are among the most common ailments in the clinic 1, 2. The treatments for healing wounds include drugs or application of bio-activators, wound dressing, and the autologous skin grafting 3-5. Applying bio-activator lacks adequate action time while skin grafting may cause trauma in the donor site. Besides, traditional wound dressings fail to efficiently regulate the healing process, which limits the progress of skin regeneration 6. Also, persistent oxidative stress hampers skin regeneration 7, stressing the need for developing novel wound dressings with the ability to regulate healing efficiently.

Reactive oxygen species (ROS) has a profound influence on the healing process as it promotes inflammatory response, cell proliferation, angiogenesis, the formation of granulation, and development of the extracellular matrix 7-9. During healing, excessive ROS contributes to reduced migration and proliferation of fibroblasts, keratinocytes, and endothelial cells. ROS activates various transcription factors including nuclear factor erythroid 2-related factor 2 (Nrf-2), nuclear factor kappa B (NF-κB), and activator protein-1 (AP-1) as well as stimulating the mitogen-activated protein kinase (MAPK) pathway which may lead to chronic non-healing wounds 10-12.

The ideal biomaterials for wound dressing must possess the ability to modulate cell differentiation and proliferation, control the attachment and migration of cells, and have anti-oxidative ability 13-17.Collagen is an extracellular biomacromolecule abundant in skin and is of great interest in regenerative medicine due to its remarkable biocompatibility, water holding capacity, and biodegradability 18-20. Also, collagen can regulate the cellular phenotype and cell-extracellular matrix interactions, as well as modify the physicochemical properties of the scaffold to improve the healing rate and quality 21. However, scaffolds containing collagen only have poor mechanical performance and may degrade within 2-3 days at the body temperature 22. Besides, collagen lacks antioxidant properties resulting in poor performance in regulating the wound environment 23.

Graphene oxide (GO) is a derivative of graphene, and functionalization of graphene sp_2_-bonded carbon to produce 2D nanomaterials has shown high potential as nanocarriers for biological molecules and therapeutic drug delivery 24-26. Due to its numerous -COOH and -OH groups and unique lamellar structure, GO can promote the mechanical properties and biological interactions when functionalized with collagen by providing more binding sites for bioactive growth factors or specific drugs 27, 28. Thus, GO may play an essential role in skin regeneration by combining with other biomaterials and drugs. For example, Fernández et al. investigated a GO-based material by combining PVA with other bioactive components that showed high potential for application in skin regeneration 29. Also, Kawamoto et al. reported that a collagen sponge coated with GO dispersion accelerated periodontal wound healing of class II furcation defects in dogs indicating its broad application prospects 30. Previously, researches showed that GO could serve as a drug carrier as well as a platform for slow release of other biomolecules. Feng et al. investigated the zwitterion-modified GO for doxorubicin hydrochloride loading 31. Ma and colleagues fabricated GO with hydrogel to create a novel adhesive injectable composite wound dressing, which showed great drug-releasing property and a high potential for skin regeneration 32.

N-acetyl cysteine (NAC) is a compound approved by the U.S. Food and Drug Administration for clinical use as a mucolytic which has thiol and carboxyl groups 33, 34. NAC may have a broader therapeutic potential, particularly in the setting of antioxidants 35. Recently, Zayed et al observed that NAC could accelerate amputation stump healing by increased neovascularization casting new light on its use in wound healing 36. Besides, NAC can reduce the ROS level of the injured area, thus protecting the functional cells from the damage caused by excessive ROS 37. Theoretically, the -COOH of NAC makes it possible to form an amide linkage with collagen and GO and enable sustained release.

**Figure 1:**
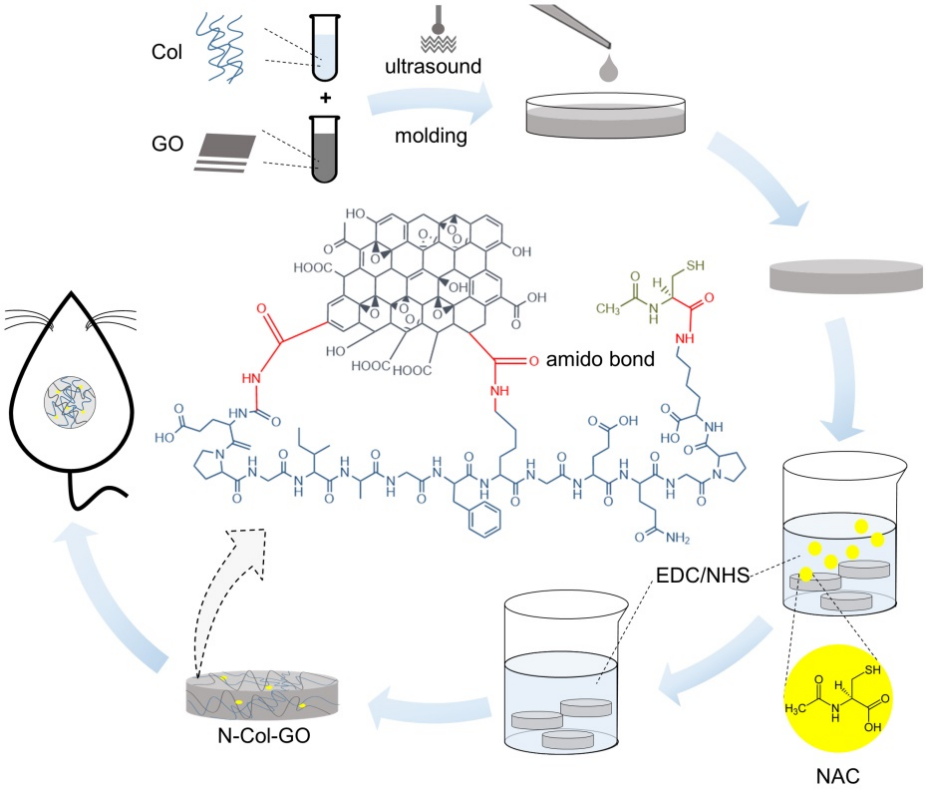
Schematic of the experimental procedure. Collagen solution (10 mg/mL) and GO solution (8 mg/mL) were thoroughly mixed under the ultrasonic wave and added into a cylindrical mold with a diameter of 20 mm and height of 2 mm. After lyophilization, NAC (0.1 mg/mL) was loaded onto the Col-GO hybrid membrane, which was then crosslinked by EDC/NHS. Subsequently, the N-Col-GO hybrid membrane was freeze-dried for the second time. Skin defects of 20 mm thickness were created on the back of 32 Male SD rats (age: 8-10 weeks; weight: 25-150 g) and repaired with N-Col-GO hybrid membrane.

In this study, N-acetyl cysteine-loaded graphene oxide-collagen hybrid membrane (N-Col-GO) was synthesized by EDC/NHS mold with a diameter of 20 mm (Figure 1). The membrane's physical properties, drug release ability, water retention capacity, surface topology, and *in vitro* biocompatibility were characterized. Finally, a rat model with a 20 mm full- thickness skin defect on the dorsal side was used to verify the skin regeneration effect of N-Col-GO.

## Materials and Methods

All chemicals used were of pure analytical grade. Type I collagen was purchased from First Link Ltd, West Midlands, UK. Other reagents were purchased from Sigma Aldrich (USA).

### Preparation of N-COL-GO hybrid membrane

GO solution (8 mg/mL) was prepared by adding 80 mg of GO in 10 mL of phosphate buffer saline (PBS) to keep the pH of the mixture at 6.5. The mixture was homogenized with a probe syndicator for 30 min with 70% intensity in an ice bath. Collagen scales (400 mg) were cut into pieces and then treated with 30 mL 0.5 M acetic acid solution over 6 h. The collagen mixture was distributed by ultra-sonic oscillation in the ice-water bath. The GO solution was then mixed with the collagen solution with a probe syndicator for 60 min with 70% intensity in an ice bath. The mixed solution was laid over a cylindrical mold with a height of 2 mm and a diameter of 20 mm at -20 °C for 12 h. After freeze-drying, the dry Col-GO mixture was crosslinked by EDC/NHS 38. To load the NAC onto the collagen hybrid membrane, 0.1 mg/mL NAC was added into the EDC/NHS crosslink agent.

Collagen only membrane (Col), NAC loaded collagen membrane (N-Col), and collagen-GO hybrid membrane (Col-GO) were acquired using the above methods.

### Physical characterization of N-COL-GO hybrid membrane

#### Morphology of the hybrid membrane

The surface of the membrane was coated with gold nanoparticles and evaluated by scanning electron microscopy (SEM) (JSM-IT300, JEOL, Tokyo, Japan) at a high voltage of 20 kV.

#### Water absorption capacity

Five specimens of all scaffolds were equally weighed as W_0_ and placed in PBS for 24 h at 37 °C. After soaking, specimens were removed from PBS and weighed to obtain W_t_. The percentage weight absorption was calculated by the following equation.


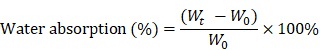


#### Water retention ability

A filter with a diameter of 0.5 mm holes was used to hold the wet samples. After centrifugation (AccuSpin 400, Fisher Scientific) at 260 g for 5 minutes at room temperature, the sample weight was measured as W_1_ and water retention was calculated as follows.


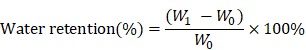


#### Porosity

Porosity was determined following the fluid saturation method using the equation below where V_b_ refers to the volume of the scaffold, while ρ_wtr_ refers to the density of water (1.00 g/cm^3^).


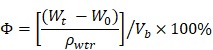


#### Elastic modulus measurement

The stretching and compressing testing system (INSTRON 5967, US) was used for elastic modulus measurement to evaluate the mechanical properties.

### Mechanical Characterizations of the Scaffolds

#### X-ray diffraction (XRD) analysis

The XRD was performed using a wide-angle X-ray scattering diffractometer (Empyrean, PANalytical B.V. Netherlands) with Cu-Kα radiation (k =0.154056nm) in the range of 5°-69° (2θ) at 40 kV and 30 mA.

#### Fourier Transform Infrared Spectroscopy (FTIR)

FTIR (VERTEX 70, Bruker, Germany) measurements were carried out at a resolution of 4 cm^-1^ in the frequency range of 4000-500 cm^-1^.

#### Raman Spectra Analysis:

The Raman spectrum (Rfs 27 FT, Bruker, Germany) was measured with a scanning range of 50-4000 cm^-1^. The excitation source was a diode laser with a wavelength of 532 nm adjusted to a power of 30 mW 39.

### *In vitro* NAC release

Three specimens of N-Col-GO and N-Col hybrid membrane were immersed in 2 mL of PBS at 37°C, then transferred into Eppendorf tubes and kept in a thermoshaker. At 1, 2, 4, 8, 24, and 48h, 7 days, and 14 days, the NAC released in the solution was separated from the N-Col, and N-Col-GO hybrid membranes by centrifuging at 3000 rpm for 5 min. The released NAC concentration was determined by high-performance liquid chromatography (HPLC) using a C18 chromatographic column (200 mm×4.6 mm, 5 μm). The mobile phase was 20 mmol/L sodium dihydrogen phosphate solution- acetonitrile (94:6) with a flow rate of 1.0 mL/min, the sample size was 10 μL, and the detection wavelength was 210 nm. The standard curve of NAC in PBS was used for calculating the concentration of released NAC.

### Biocompatibility assay

NIH 3T3 fibroblasts (ATCC, America) were used for cytology experiments. Dulbecco's Modified Eagle Medium (DMEM) supplemented with 10% fetal bovine serum, 5% penicillin and streptomycin was used for cell maintenance. The medium was replaced every 3 days. The cells were incubated at 37°C with 5% CO_2_. The cells were detached using 0.05% trypsin 40. Specimens of ethylene oxide sterilized Col, N-Col, Col-GO, and N-Col-GO hybrid membranes were added to each culture plate and the plates were washed with sterile PBS for 1 hour. Each membrane was seeded with a concentration of 5×10^3^ cells and the membranes were incubated with the complete medium. The cell biocompatibility assay was performed as described below.

#### Live/dead cell staining

Fluorescein diacetate/propidium iodide (FDA/ PI) staining was performed for detecting live and dead cells in the scaffold on d1, d3, d7, and d14 of culture as previously described 40. The cytoplasm of viable cells was stained green by the FDA and the necrotic and apoptotic cell nuclei were stained red by PI. The fibroblasts seeded Col, N-Col, Col-GO, N-Col-GO hybrid membranes were then observed under a confocal laser microscope (Nikon Ti, Japan) operated at 200 kV.

#### SEM analysis

Membranes seeded with NIH 3T3 fibroblasts for 7 days were used for SEM analysis. After the culture media was removed, the membranes were washed with PBS and fixed using 4% paraformaldehyde. The membranes were then dehydrated using graded ethanol, dried for 8 hours and observed by SEM according to the above procedure.

#### MTT assay

The adherence and proliferation of fibroblasts were visualized and quantified by MTT assay according to the previously described method 41.

#### Cell migration assay

The cell migration assay was performed as previously described 42. NIH 3T3 fibroblasts were seeded in a 6-well plate with a density of 2.5×10^5^ cells in each well and cultured until 80% to 85% confluence was reached. A scratch was made by a sterile 200 mL tip. The wells were then rinsed with PBS gently for 3 times and refilled with DMEM containing a Col, N-Col, Col-GO, and N-Col-GO hybrid membrane. The area of the remaining spaces was measured by Image J software at 0, 8, 16, 24 h of incubation.

### *In vivo* test on rat wound healing model

All procedures were performed in accordance with the guidelines approved by the Animal Ethical Committee of Huazhong University of Science and Technology. 32 Male rats (age: 8-10 weeks; weight: 25-150 g; SD) were randomly divided into 4 groups and housed individually in standardized environmental conditions. After being anesthetized with the intraperitoneal injection of pentobarbital sodium (30 mg/kg, Sigma-Aldrich, America), the hair of the back of the rats was shaved, and subsequently swabbed with betadine. A 20 mm diameter incision was made on the center of the dorsal side of all the rats, and the wound area was sterilized with 1% polyvinylpyrrolidone. The incisions were then covered with Col, N-Col, Col-GO, or N-Col-GO hybrid membranes, which were fixed on the wound area using a bandage. Regular dressing changes were carried out for all the rats on alternate days.

### Percentage of wound closing area

At d1, d3, d7, and d14, the wound closure was measured by the percentage reduction in wound area. The wound area was photographed by a fixed camera tracking the wound margin on a 10 cm scale. Subsequently, the wound reduction area was measured using Image J software. Percentage of wound closure was then calculated by the formula below.


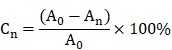


Where C_n_ is the percentage of wound area reduction at d1, d3, d7, and d14 post wounding, A_0_ is the original wound area, and A_n_ represents the wound area at d1, d3, d7, d14 post wounding.

### Histologic analysis

Tissues collected 14 days post-operative were fixed and stained with Hematoxylin-Eosin (H & E) as well as Masson trichrome stains. An Olympus IXplore microscope was used for observation and measurement.

### IHC examinations

The tissues were immunocytochemically stained with antibodies against human-specific CD31 (ARG52748, Arigo). Immunoreactivity was visualized using an isothiocyanate-conjugated secondary antibody (Jackson Immuno Research Laboratories Inc., West Grove, PA, USA) and imaged using an optical microscope (Olympus BX53).

### RT-PCR analysis

The relative mRNA expression of various genes was determined by RT-PCR analysis. All RT-PCR reactions were performed using a Stepone Plus real- time PCR system (ABI) with an ultramicrospectrophotometer (NanoDrop2000, Thermo). Each cycle consisted of the following temperatures: 94 °C for 3 s and 60 °C for 30 s. The primer sequences used are shown in supporting information as Table S1.

### Statistical analysis

Quantitative results were expressed as the mean value from at least triplicate samples ± standard deviation. Statistical analysis was performed using one-way ANOVA and Student's t-test (SPSS software 13.0, USA; GraphPad Prism 7.0, USA). A value of p < 0.05 was considered to be statistically significant.

## Results

### Physical characterization of N-Col-GO hybrid membrane

**Figure 2:**
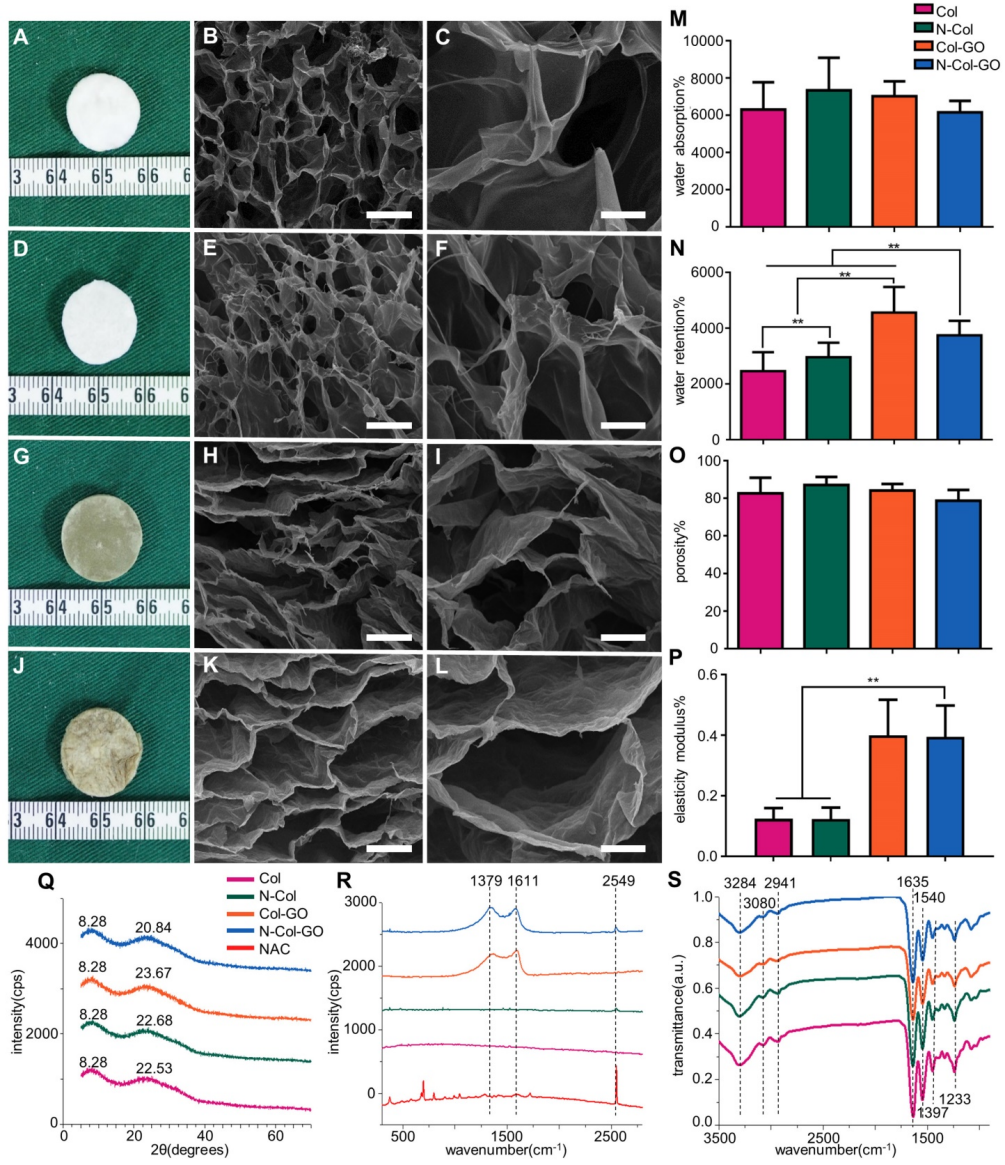
Characterizations of N-Col-GO hybrid membrane. Macroscopic view (A, D, G, J) and SEM images (B-C, E-F, H-I, K-L) of the Col, N-Col, Col-GO, and N-Col-GO hybrid membranes. Water absorption (M), water retention (N), porosity (O), elasticity modulus (P), and X-ray diffraction of N-Col-GO hybrid membrane (Q). (R) Ramon spectrum of N-Col-GO hybrid membrane; dashed lines show the specific peak of GO at 1379 nm and 1611 nm, while the specific peak of NAC is at 2549 nm. (S) (ATR)-FTIR of N-Col-GO hybrid membrane shows major peaks of collagen, GO, and NAC. (mean ± SD; *:P < 0.05, **:P< 0.01; Scale bars in B, E, H, and K: 100 µm; C, F, I, and L: 20 µm.)

We observed that membranes with Col, N-Col, Col-GO, and N-Col-GO hybrid had a different appearance. The collagen only dispersion was a semi- transparent white color sponge, and so was the N-Col hybrid membrane. The surface morphologies of Col, N-Col, Col-GO, and N-Col-GO hybrid membranes are shown in Figure 2A, D, G, J.

The water absorption ratio of the native collagen hybrid membrane was 6302.94±1467.478% while for the N-Col, Col-GO, and N-Col-GO hybrid membranes, the ratio was 7336.560±1750.120%, 7024.19± 795.921%, and 653.64±619.444%, respectively, with no significant difference (Figure 2M). Figure 2N shows that the water retention ability of N-Col-GO (3750.00 ±521.800%) and Col-GO membranes (4569.20± 920.289%) was significantly higher than that of the Col membrane (2468.05±680.375%) and N-Col membrane (2965.68±522.043%) due to the incorporation of GO.

Compared to the native collagen membrane with a porosity of 82.51±8.375%, the N-Col, Col-GO, and N-Col-GO hybrid membranes also showed high porosity of 87.00±4.279%, 84.05±3.471%, and 78.58± 5.818%, respectively, as shown in Figure 2O. The pore structures of Col-GO, and N-Col-GO hybrid membranes were better-distributed and interconnected than the Col and N-Col hybrid membranes. The conjugations of NAC with Col or Col-GO hybrid membrane had no influence on the scaffold pore size as shown by the SEM images (Figure 2). It was evident that the interconnecting pores covered most of the space in the membranes. The high porosity revealed the applicability of the N-Col-GO membrane for biomedical use.

The representative elastic modulus of the composites is shown in Figure 2P. The elastic moduli of Col and N-Col membranes were found to be 0.124 MPa and 0.123 MPa, respectively. A significant improvement could be observed in the elastic modulus (0.400 MPa and 0.395 MPa) with the addition of GO.

Figure 2Q shows the XRD of Col, N-Col, Col-GO, and N-Col-GO membranes. The peak with the highest intensity of collagen was at 2θ=22.53°, and its d spacing was 3.94 Å, while the Col-GO spectrum showed the most intense peak at 2θ=23.67° with a d spacing of 3.73 Å. After loading with NAC, the peak and d spacing did not change much. The N-Col hybrid membrane had a peak at 2θ=22.69°and d spacing of 3.91 Å, while N-Col-GO hybrid membrane had a peak at 2θ=23.84°and d spacing of 3.73 Å. All four types of scaffolds exhibited a characteristic peak around 2θ=8.28°. The broad peak represented a typical diffraction pattern of collagen. A broad spectrum at 2θ=23.84° displayed by the diffraction spectrum of N-Col-GO and Col-GO membranes showed their amorphous nature. The intermolecular distance of the scaffold hybrids with GO was observed at 3.73 Å which was slightly smaller than that of the Col membrane or N-Col membrane (3.94 Å) which could be due to the aggregation of GO with Col. The loaded NAC did not influence the crystal structure of these scaffolds.

Raman spectroscopy provided helpful information on the structural characteristics of N-Col-GO hybrid membrane. Raman spectra of the samples are shown in Figure 2R. The spectra of N-Col-GO hybrid membrane showed three evident symbols: G band (1661 cm^-1^), D band (1379 cm^-1^), and -SH stretching (2549cm^-1^) and were derived from in-plane vibrations of sp_2_ carbon atoms of the graphite lattice. D band was due to the out-of-plane breathing mode of the sp_2_ atoms. These observations confirmed the layer separation of the GO structure. Also, the corresponding -SH stretching band indicated that NAC was loaded in the N-Col-GO hybrid membrane 39.

FTIR characterization was used for investigating the covalent amide bond (-CONH-) interaction between GO, Col, and NAC. The FTIR spectrum of Col, N-Col, Col-GO, and N-Col-GO membranes are shown in Figure 2S. The results of Col and Col-GO membranes were consistent with previous reports 43. In the spectrum, the -NH_2_ stretching vibration was detected as the peak at 3284 cm^-1^, while the Fermi resonance overtone of the 1540 cm^-1^ band responded to 3080 cm^-1^. The peak of C-H stretching was at 2941 cm^-1^. 1635, 1540, and 1397 cm^-1^ bands were assigned to C=O stretching, N-H bonding, and C-N stretching of amide (-CONH_2_) linkages in collagen. The C-N stretching of amine corresponded to the peak at 1233 cm^-1^. No additional peaks were noticed after comparing the spectra of the four types of scaffolds.

### *In vitro* drug release

**Figure 3:**
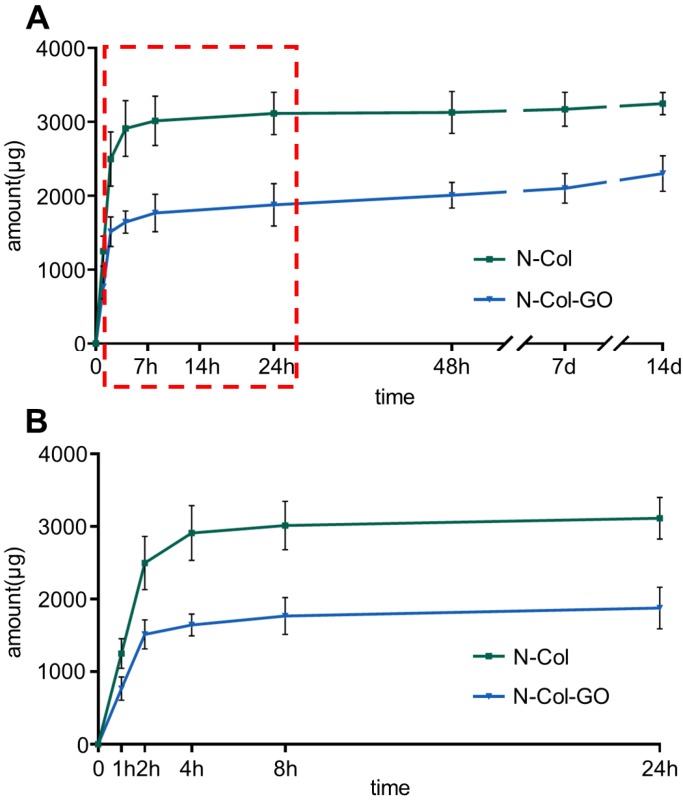
NAC release curve of N-Col-GO hybrid membrane after 14 d (A) and 24 h (B). The green curve indicates that most of the NAC, up to 3114.937±287.675 μg, was released within 24 h in N-Col membrane. The blue curve shows that the N-Col-GO hybrid membrane released 1877.837±287.617 μg of NAC within 24 h followed by sustained release up to 2302.462±239.754 till 14 d indicating that N-Col-GO had a better drug release ability.

The release profiles of NAC from N-Col and N-Col-GO groups are shown in Figure 3. The release of NAC from both scaffolds was rapid for the first four hours, namely 2910.448±377.457 μg in the N-Col and 1643.396±151.405 μg in N-Col-GO groups. When immersed in PBS for 24 hours, the NAC amount was 3114.937±287.675μg, 1877.837±287.617μg respectively, indicating that the release of NAC in N-Col-GO group was much slower than that in the N-Col group. Surprisingly, nearly all the loaded NAC in the N-Col group was released in 24h after being immersed in PBS, while NAC in N-Col-GO exhibited sustained release during 14 days. During the period from 24 hours to 14 days, only about 132.575 μg NAC was released by the N-Col group while 424.625 μg NAC was released by the N-Col-GO group.

### Biocompatibility of N-Col-GO hybrid membrane *in vitro*

**Figure 4:**
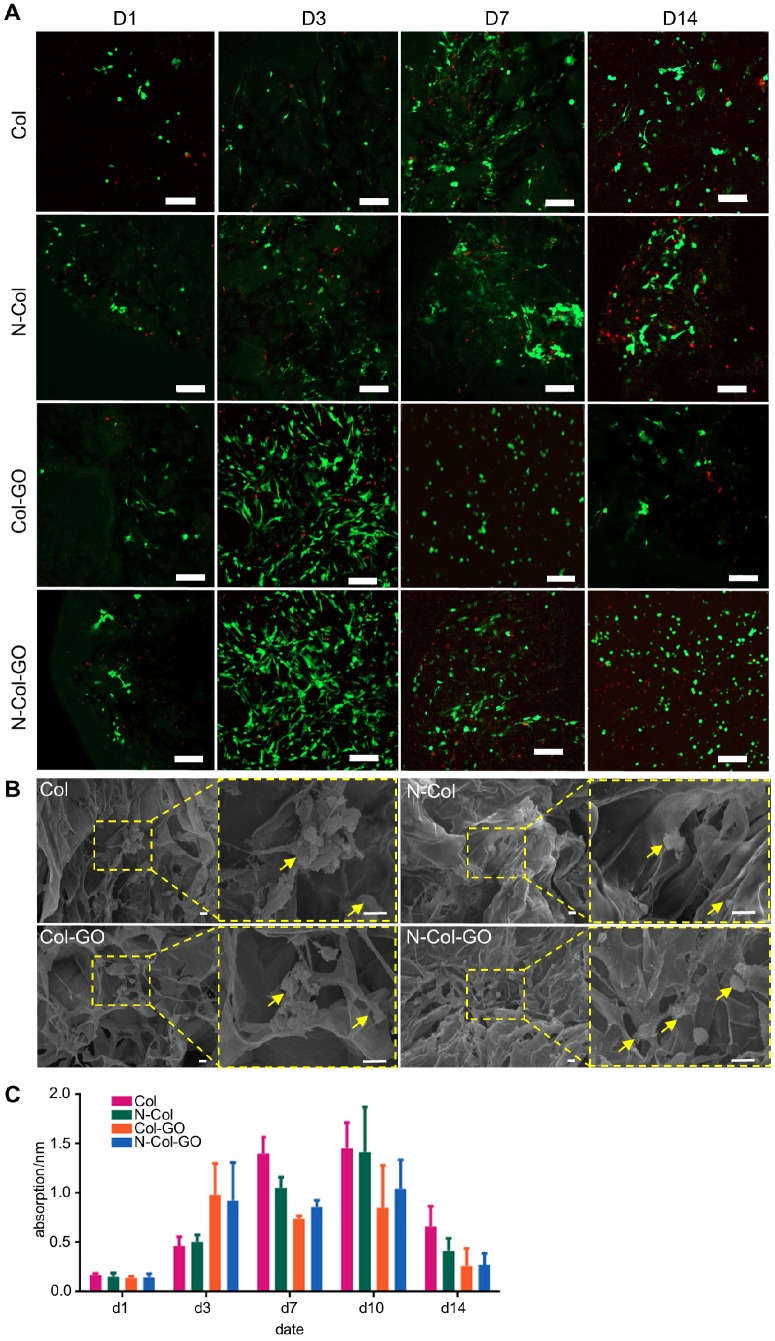
Biocompatibility of N-Col-GO hybrid membrane. (A) FDA/PI staining of N-Col-GO hybrid membrane seeded with NIH 3T3fibroblasts observed on day 1, 3, 7, and 14. (B) SEM image of N-Col-GO hybrid membrane seeded with NIH 3T3fibroblasts observed on day 7. The yellow arrow indicates the fibroblasts adhered on the membranes. (C) MTT assay of NIH 3T3 fibroblasts seeded onto N-Col-GO hybrid membrane performed on different days. (mean ± SD; Scale bars in A: 200 µm; B: 10 µm.)

Live and dead cell staining of scaffolds with fibroblasts after 1, 3, 7, and 14 days are shown in Figure 4A. When the fibroblasts were stained with calcein fluorescent probe, live cells released green fluorescence caused by FDA staining while dead cells released red-fluorescence caused by PI. In the present study, a slight increase was seen in FDA fluorescence intensity in the N-Col-GO and Col-GO membrane- treated cells compared to the control group on day 3. The number of cells decreased during day 7 to day 14 in the GO group which may be related to the toxicity of GO. However, the cell viability of N-Col-GO group was better compared to the groups without NAC. Furthermore, SEM on day 7 (Figure 4B) showed comparable results with the FDA/PI staining. The cells on N-Col-GO membrane were more spindle- shaped while the ones on Col-GO displayed spherical dead shapes. The fibroblasts adhered on the membranes as indicated by the yellow arrow.

The MTT results (Figure 4C) indicated that treatment with Col exhibited excellent cell viability for 7 days and showed slightly higher cell proliferation rate compared to the control. Col-GO-treated cells showed similar cell viability with a noticeable decrease in cell proliferation after 7 days, whereas the presence of NAC improved the cell viability slightly. The number of cells increased with increasing culture time in all groups for 10 days after seeding.

**Figure 5:**
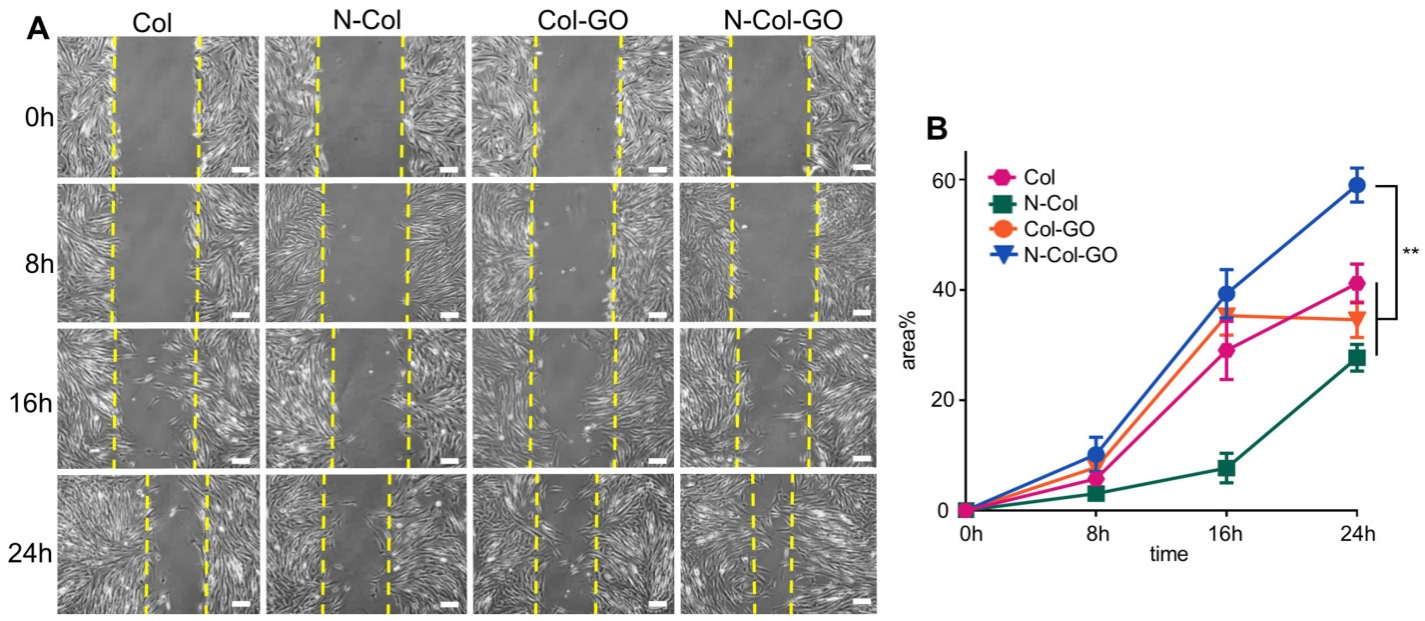
Scratch test of NIH 3T3 fibroblasts. (A) Images of the scratch test of fibroblast cultured by leaching solution of N-Col-GO hybrid membrane after 0, 8, 16, and 24 h observed under a light microscope. (B) Immigrated area of fibroblast cultured by leaching solution of N-Col-GO hybrid membrane after 0, 8,16, and 24 h. (mean ± SD; *:P < 0.05, **:P< 0.01, Scale bars in A: 100 µm)

Results of the cell migration assay using NIH 3T3 fibroblasts showed that N-Col-GO had the fastest fibroblast migration rate of 39.3%±4.38% compared with Col-GO (35.3±3.53%), N-Col (7.70±2.67%) and Col (29.1±5.31%) groups in 16 h. Compared to the Col (41.2±3.52%), N-Col (27.7±2.40%) and Col-GO (34.6±3.22%) membranes, N-Col-GO (59.0±3.10%)-treated cells migrated at the fastest rate with nearly 60% of the scratch area being covered in 24h (Figure 5).

### *In vivo* wound healing experiments

**Figure 6:**
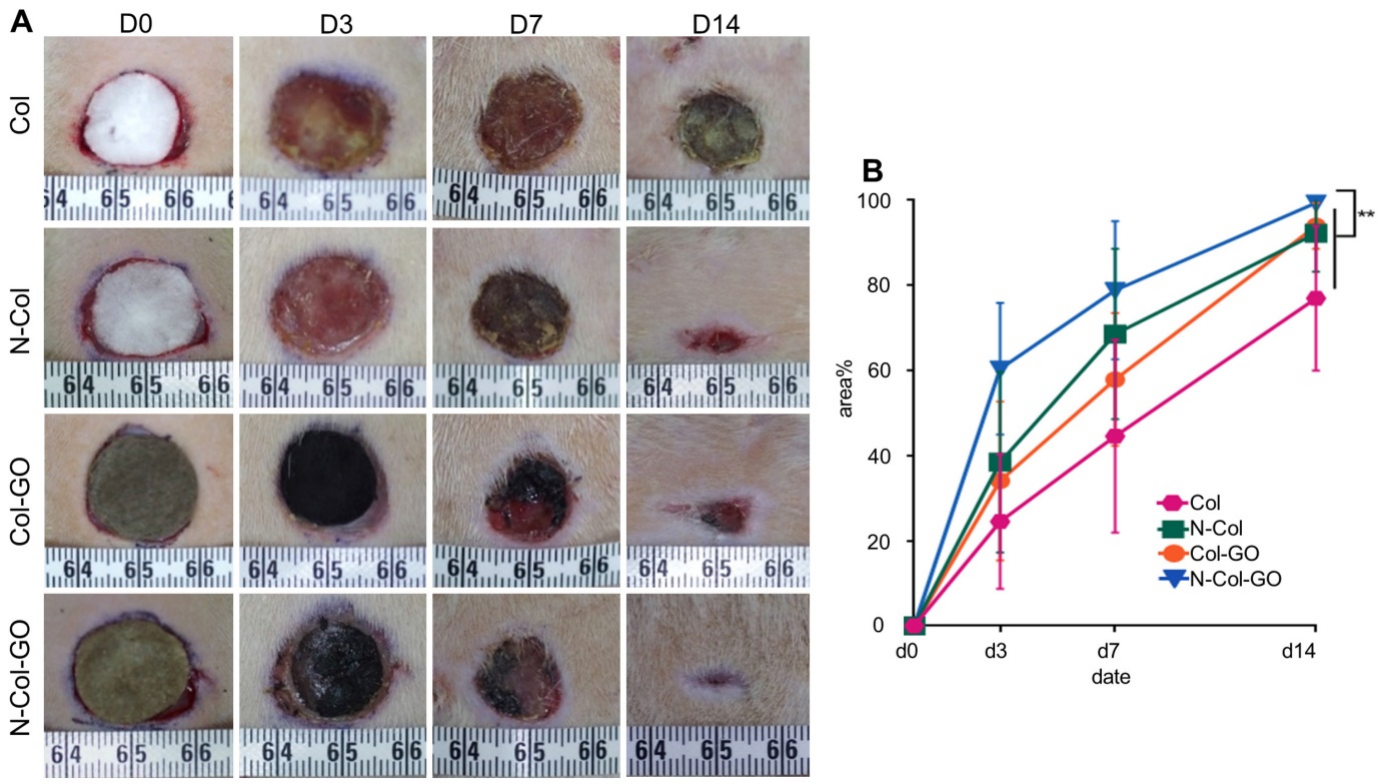
20 mm dermal defect repaired by Col, N-Col, Col-GO, and N-Col-GO hybrid membranes in rats. (A) Photographic evaluation of skin regeneration at day 14. N-Col-GO has completely healed while other groups failed. (B) Area ratio of rat wound healing. N-Col-GO group achieved the best therapy effect at three different time points (3, 7, and 14 days) compared with other groups. (mean ± SD; *:P < 0.05, **:P< 0.01, n = 8 per group).

To compare the wound healing efficiency, Col, N-Col, and Col-GO membranes were used as controls. Among the four groups, N-Col-GO membrane showed the highest wound closure rate (Figure 6A-B). The N-Col-GO membrane adhered well on the wound surface in the first 7 days, and later the scaffold was embedded subcutaneously. After 7 days of treatment, the percentage of wound closure was around 78.9±16.26% for the N-Col-GO membrane whereas Col, N-Col, and Col-GO membranes exhibited 44.6±22.63%, 68.6±20.02%, and 57.9±15.60% wound closure, respectively. On day 14, all rats with N-Col-GO membrane presented a complete wound closure (99.4±1.14%), whereas the wound closure was 77.0±17.00%, 92.2±9.01%, and 94.00±5.38% for Col, N-Col, and Col-GO membranes, respectively.

### Histologic examination

**Figure 7:**
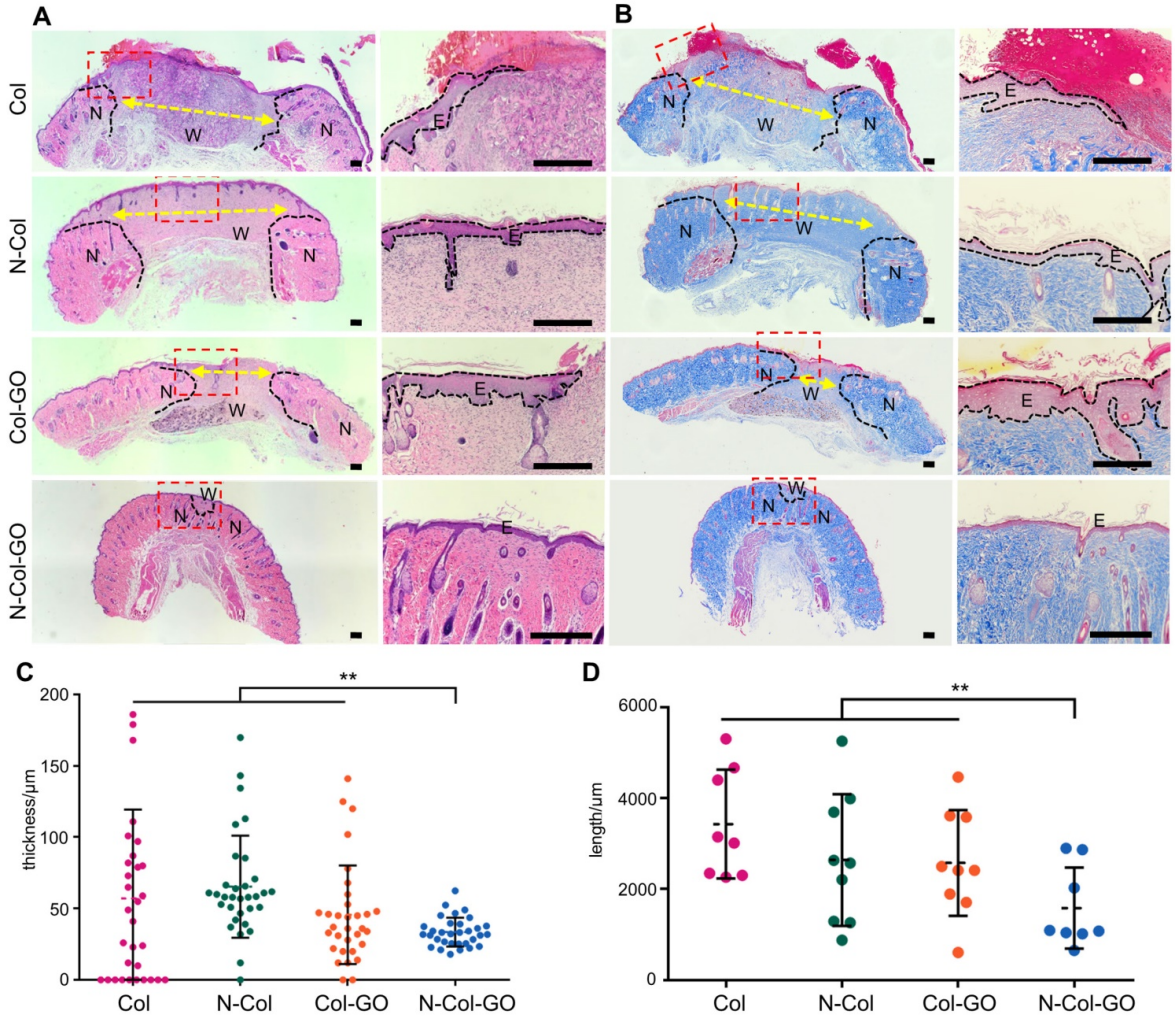
Histological analysis of wound areas on day 14. (A&B) H&E staining and Masson staining of tissue sections. The dotted line is the interface between the wound area and neo-epidermis. Yellow arrows indicate remaining wound length; W, wound area; N, neo-epidermis; E, epidermis. Scale bar = 200 μm. (C) Thickness of regenerated epidermis. (D) Length of the remaining wound. Data represent means ± SD (n = 8) (*:P < 0.05, **:P< 0.01)

H&E stained sections of healing areas treated with four types of scaffolds are shown in Figure 7A. After 14 days, newly formed granulation tissue in the collagen-treated area showed severe inflammatory response with the aggregation of neutrophils and basophils. The N-Col-treated group presented fewer neutrophils and macrophages along with fewer fibroblasts. The Col-GO-treated group also showed fewer neutrophils and macrophages but more fibroblasts. N-Col-GO treated group, on the other hand, displayed the least inflammatory response when compared with the other groups showing few neutrophils and macrophages. However, the number of fibroblasts observed in this stage for N-Col-GO- and Col-GO- treated groups was slightly less, especially around the area where GO was not yet absorbed.

'

On day 14, the N-Col-GO-treated group reached complete epithelialization with the well-developed dermis and thick epidermis. Moderate epithelialization with very thin epidermis was observed in the N-Col group, as well as little granulation tissue formation, whereas the Col-GO-treated group showed partial healing with better but in-connective granulation formation. However, unhealed wounds with poor epithelialization and little granulation tissue formation were observed in the Col group. The average epidermis thickness of all wounds is displayed in Figure 7C. The N-Col-GO-treated group (33.5±10.10μm) had the best epidermal maturation with uniform thickness while Col-treated group (57.1±62.21μm) exhibited incomplete epithelization with uneven thickness.

Notably, in the N-Col-GO-treated group, epidermal layer along with dermal layer, sweat glands, as well as hair follicles were present which indicated that the incisions had passed the remodeling phase and reached complete skin regeneration in the healing process.

Collagen formation is of great significance in tissue reconstruction. Masson trichrome staining of the healing area after 14 days is displayed in Figure 7B. On day 14, the N-Col-GO-treated group presented a uniform and thick collagen bundle deposition showing complete wound healing while the number of collagen fibers was less and non-uniform in control groups. Due to insufficient collagen deposition, an unreconstructed area which was stained as white space was observed in the dermis of all groups both in HE and Masson staining. Statistics of the average length of unreconstructed area in Figure 7D shows that the N-Col-GO-treated group (1589±890.1 μm) enhanced wound healing by formation of more granulation, while in Col, N-Col, and Col-GO membrane-treated groups, the unreconstructed areas were 3426±1197 μm, 2641±1447 μm, 2576±1162 μm, respectively, which could be caused by the increase in the number of fibroblasts.

### IHC examination

**Figure 8:**
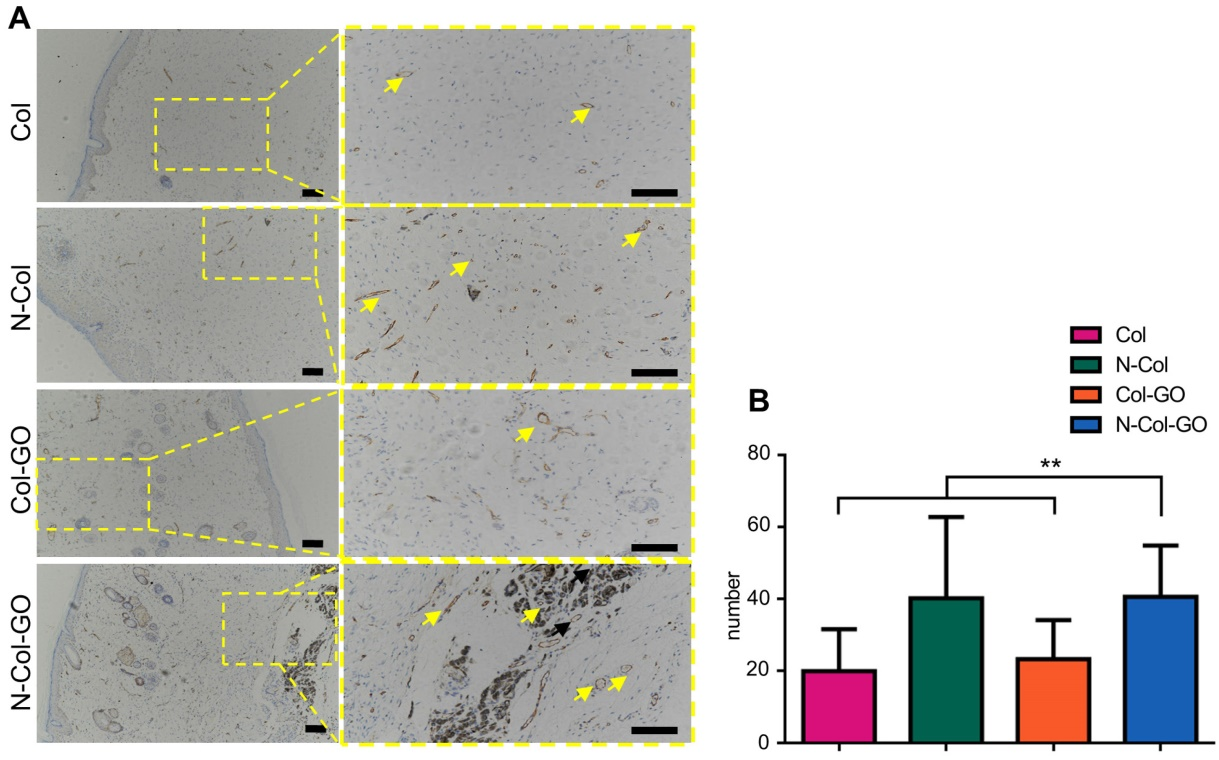
CD31 IHC examination of regenerated skin on day 14. (A) CD31 staining of tissue sections. Yellow arrows indicate neovascularization. Scale bar = 200 μm. (B) Number of blood capillaries. Data represent means ± SD (n = 8) (*:P < 0.05, **:P< 0.01)

The number of blood capillaries were counted in all four groups (Figure 8A-B). CD31 showed the least number of blood capillaries 20.0±11.69 in the Col- treated group and a comparable number, 23.4±10.82, of capillaries in Col-GO-treated group. N-Col and N-Col-GO membrane-treated groups almost doubled the number of blood capillaries to 40.3±22.63 and 40.7±14.27, respectively, which suggested that NAC significantly increased angiogenesis during wound healing.

### RT-PCR analysis

**Figure 9:**
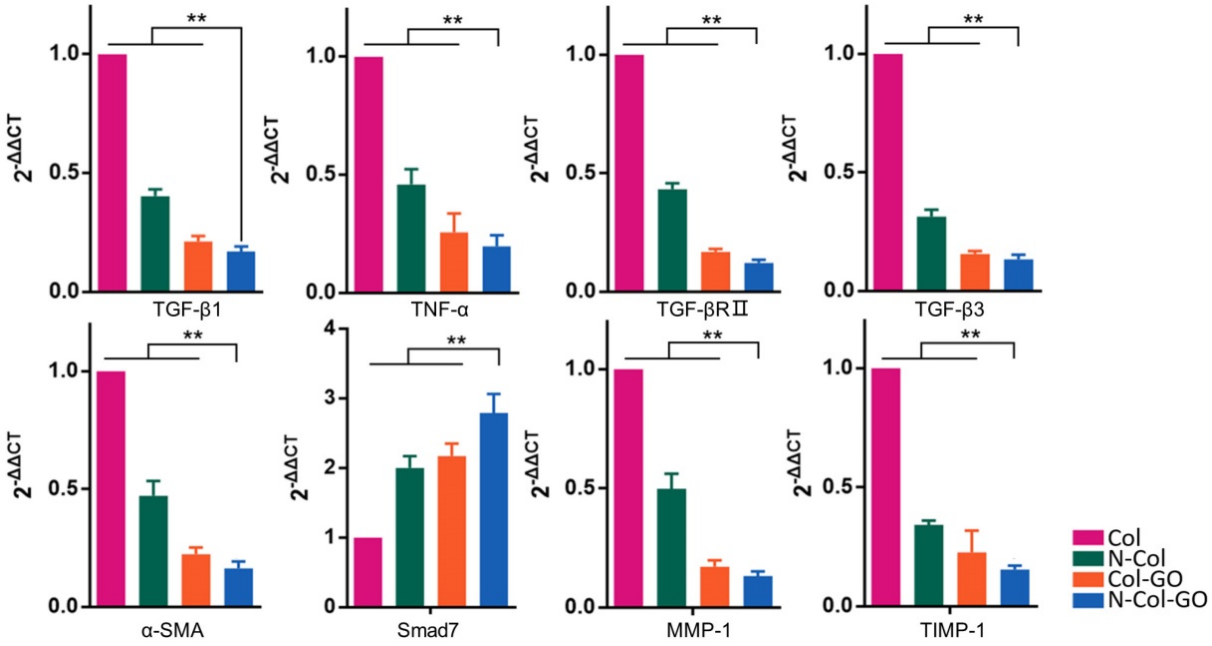
Relative mRNA expressions of TGF-β1, TNF-α, TGF-βRⅡ, TGF-β3, α-SMA, Smad7, MMP-1, TIMP-1 14 days post-wounding. The N-Col-GO treatment induced significantly lower mRNA expression of profibrotic factors, including TGF-β1, TNF-α, TGF-βRII, TGF-β3 and α-SMA, and overexpression of anti-fibrotic factors, including inhibitory Smad7 protein. Expression of TIMP-1 and MMP-1 was downregulated and upregulated, respectively, indicating that the collagen metabolism disorder has been controlled. Data represent means ± SD (n = 8) (*:P < 0.05, **:P< 0.01)

The mRNA expression of several factors involved in wound healing and later scar formation was examined by RT-PCR. The results indicated that at 14 days post-wounding, compared with other groups, Col-GO and N-Col-GO-treated groups led to remarkably lower mRNA levels of profibrotic factors, including TGF-β1, TNF-α, TGF-βRII, TGF-β3, and α-SMA 44, 45, and a significantly higher level of anti-fibrotic factors, including inhibitory Smad7 protein (Figure 9). Moreover, mRNA expression of TIMP-1 and MMP-1 was separately down- and upregulated, respectively (Figure 9), indicating that the collagen metabolism disorder had been controlled.

## Discussion

Regeneration of full-thickness of the skin after injuries has always been a problem. In this study, we present a simple and efficient technique to fabricate a collagen-based membrane by hybrid GO-containing antioxidant NAC. The designed technique leveraged on the inherent advantages of GO and NAC to enhance skin regeneration. The NAC-loaded GO- collagen hybrid membrane displayed excellent water retention capacity, porosity, biocompatibility, and the sustained drug-releasing property. In a mouse model of full-thickness skin injuries and *in vitro* experiments, we demonstrated that the N-Col-GO hybrid membrane accelerated fibroblast migration and adhesion, promoted epidermis maturation, and enhanced angiogenesis, leading to rapid skin regeneration. Furthermore, at the end of the healing procedure, the gene expression of profibrotic factors and collagen deposition was down-regulated indicating successful scarless healing.

An ideal scaffold for wound healing is expected to have high water retention capacity, biocompatibility to facilitate fibroblast and keratinocyte infiltration, promote proliferation, form reparative tissues, and possess anti-oxidative capacity 46, 47. The water retention capacity of a scaffold is closely associated with its hydrophilic nature and microstructure. Because the GO (abundant -COOH and -OH groups) and collagen (-NH_2_ and -COOH groups) are both hydrophilic materials, the Col-GO, N-Col-GO membranes have high water retention capability with considerable mechanical properties as shown in Fig 2O. Also, GO has high biocompatibility and is a good substrate for regenerative medicine due to its physicochemical properties such as large specific surface area, good dispersibility, and hydrophilicity 48. The effectiveness of GO as a skin regeneration material was investigated by Hanping et al. They used polyethylene glycol-coated graphene oxide (GO-PEG) for skin repair and reported that GO-promoted collagen deposition and angiogenesis in diabetic wound repair 49 which is consistent with our observations in the mouse full-skin defect model (Figure 6). However, the biotoxicity of GO is controversial based on previous studies. For instance, Bing and coworkers reported that GO had low toxicity on cell cytoskeletal organization in Raw264.7 mouse monocyte-macrophage cells 50. Wierzbicki et al. also found that GO did not exert strong toxicity, although it effectively deregulated cell migration and adhesion 51. These findings are in agreement with our results of the influence of GO on fibroblasts as shown in Figure 5B. At 16 h, the migration rates of the Col and Col-GO and groups were similar. However, the migration of Col-GO group stopped in the following 8 h. Two studies by Vázquez et al. and Macosko et al reported that the cytotoxicity of GO was preceded by increased ROS levels in cells 52, 53. Our results showed that reduced toxicity of GO was due to NAC, which was incorporated into the Col-GO membrane, enabled its persistent release, and down-regulated the level of ROS induced by GO. As shown in Figure 5B, the fastest migration rate was observed in N-Col-GO group compared to Col-GO and other groups indicating that incorporating the antioxidant into the GO reduces the cytotoxicity of GO and increases cell migration and adhesion.

Finally, the high anti-oxidative property of the novel membrane was due to the ability of GO to interact with other biological molecules through its functional groups, which makes GO an effective carrier for the antioxidant NAC. NAC forms an amido linkage with the hydroxy groups on collagen and GO, enabling it to be loaded into Col-GO membrane for sustained drug-release as shown in Figures 1&3.

One of the main objectives of regenerative treatments is to lower the ROS level generated in the inflammatory phase of healing, which may last for several days. However, NAC loaded into the Col membrane did not effectively lower the inflammation level due to decreased NAC release after 24 hours. On the contrary, N-Col-GO membrane could sustain the release of NAC for more than 14 days which not only lowered the ROS level in the inflammatory phase but also promoted angiogenesis, granulation, tissue formation, and epithelialization during wound healing. The sustained release of NAC can be categorized into two phases. During the first phase, rapid release occurs in the first couple of hours due to the release of free NAC deposited inside the pores of collagen or Col-GO. This type of release is rapid and occurs within 24 h. The cumulative release of NAC occurs in the second phase, which is attributed to the numerous -COOH and -OH groups in GO enabling the GO-NAC interaction via amido bond. Due to the complex microenvironment in the wound, breakage of chemical bonds together with the degradation of membranes leads to the release of more NAC. Furthermore, strong NAC binding may enhance the hydrophobicity of the scaffold via -SH groups decreasing water penetration (Figure 2M-N) and retarding the diffusion of NAC from the collagen and GO matrix into the surrounding medium. On the contrary, the release rate of N-Col much faster than that of N-Col-GO group as collagen might be degraded within 2-3 days at the body temperature (Figure 3).

NAC has been reported to enhance cytocompatibility of materials, down-regulate inflammation, and promote vascularization. For instance, Zhiyong et al. found that the ability of NAC to scavenge ROS enhanced cytocompatibility by attenuating cell apoptosis and improving bone ingrowth 54. Lantos et al. postulated that NAC might down-regulate inflammation by increasing GSH and PSH levels and decreasing IL-5, IL-8, IL-10 levels in the damaged skin caused by burns, which promoted the recovery process 55. Felipe et al. reported that NAC might decrease oxidative damage and inflammation and increase angiogenesis under hypoxic conditions by regulating VEGF and HIF levels. NAC-loaded GO-Col hybrid membrane promoted vascularization, faster epidermis maturation, and displayed antioxidant and anti-inflammation effects which accelerated wound healing (Figures 7&8).

**Figure 10:**
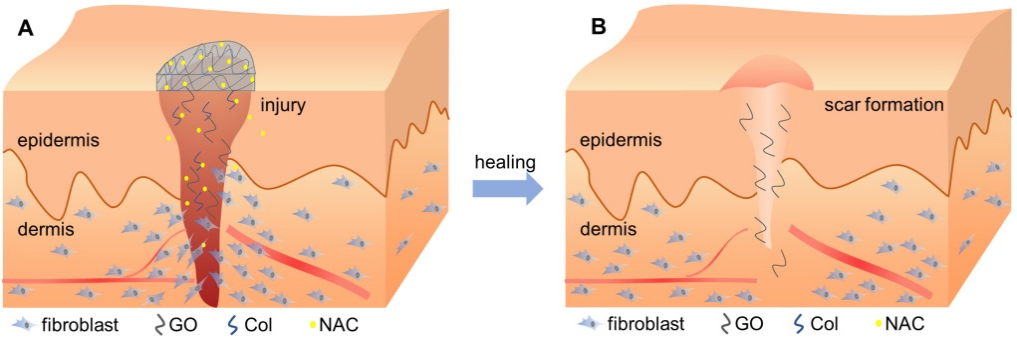
Schematic diagram of the healing procedure of N-Col-GO in wound model. (A) Sustained release of NAC by the N-Col-GO membrane reduces the cytotoxicity of GO and leads to increased migration and adherence of fibroblasts in the early phase of injury and accelerates wound healing (B) Following NAC released, GO inhibits the fibroblasts from overactivation, thus altering matrix remodeling and preventing hypertrophic scar formation in the terminal phase.

Interestingly, incorporation of GO reduced the gene expression associated with fibrosis, collagen metabolism, and scarring, but increased the expression of anti-fibrosis genes after 14 days (Figure 9). Wound healing is a complex and dynamic process, which can be divided into overlapping inflammatory, fibroblastic, and remodeling phases 56, 57. Formation of granulation tissue is the central event during the fibroblastic phase, which involves fibroblast activation, fibrosis, and collagen deposition. In the remodeling phase, various enzymes assist in removing excess collagen and decelerating fibrosis and collagen formation to create a balance between the formation of new collagen and removal of old collagen 58. As has been previously described, overactivation of fibroblasts may promote scar formation, thereby increasing fibrosis and collagen metabolism disorders 59, 60. However, Christy et al. 53 and Bing et al. 50 reported that 1% of GO may inhibit the migration and adhesion of fibroblasts by increasing ROS levels. In this study, the N-Col-GO membrane enhanced the sustained release of NAC as an antioxidant to reduce GO cytotoxicity, which improved the migration and adherence of fibroblasts in the early phase due to reduced ROS levels. These effects accelerated the wound healing process (Figure 10A). After the pre-loaded NAC was released, the presence of GO inhibited the fibroblasts from overactivation (Figure 10B), thus altering matrix remodeling and preventing hypertrophic scar formation in the terminal phase of wound healing. These results demonstrated the potential of GO in preventing scar formation.

## Conclusions

We have demonstrated that NAC loaded into GO-functionalized collagen membranes promoted rapid and scarless repair of a 20 mm rat skin defect. This was due to its strong mechanical properties and controlled release ability of NAC, which reduced the level of reactive oxygen species *in situ*.

## Supplementary Material

Table S1.

## Abbreviations

NAC: N-acetyl cysteine
GO: graphene oxide
Col: collagen
ROS: reactive oxygen species
Nrf-2n: nuclear factor erythroid 2-related factor 2
NF-кB: nuclear factor kappa B
AP-1: activator protein-1
MAPK: mitogen-activated protein kinase
EDC: 1-Ethyl-3-(3-dimethylaminopropyl)-carbodiimide
NHS: N-hydroxy succinimide
N-Col: N-acetyl cysteine loaded collagen
Col-GO: collagen- graphene oxide hybrid membrane
N-Col-GO: N-acetyl cysteine loaded graphene oxide-collagen
XRD: X-ray diffraction analysis
FTIR: Fourier Transform Infrared Spectroscopy
DMEM: Dulbecco's modified Eagle medium
PBS: Phosphate buffered saline
FDA: fluoresce in diacetate
PI: propidium iodide
