# Supplementary material for: N-acetyl cysteine-loaded graphene oxide-collagen hybrid membrane for scarless wound healing
Source: Theranostics. 2019 Aug 12;9(20):5839–53. doi: 10.7150/thno.34480 (PMC6735368; doi:10.7150/thno.34480)
Supplement: Supplementary file 1 — Table S1. [file thnov09p5839s1.pdf]

Table S1 Primer sequences of Rt-PCR

Gene	Primer
GAPDH	Sense 5'-CCATTTCCAAGACTGACAGC-3'
	Anti-sense 5'-TCATTTAGGCCAGCCCTTAC-3'
TNF-α	Sense 5'-GAGATGTGGAACTGGCAGAG-3'
	Anti-sense 5'-CACol-GOAGCAGGAATGAGAAGA-3'
TGF-β1	Sense 5'-TTTAGGAAGGACCTGGGTTG-3'
	Anti-sense 5'-CAGACAGAAGTTGGCATGGT-3'
TGF-βRII	Sense 5'-GATTTGACCTGTTGCCTGTG-3'
	Anti-sense 5'-GTATCTCGCTGTTCCCACCT-3'
TGF-β3	Sense 5'-GGGTTCACACATGAAGATGC-3'
	Anti-sense 5'-GGTGCAAGTGGACAGAGAGA-3'
MMP-1	Sense 5'-GAGTCCATGTTCATGGTGGA-3'
	Anti-sense 5'-TTGGGAAGAGCTCCTCTGTT-3'
TIMP-1	Sense 5'-CTGGTAGCCCTTCTCAGAGC-3'
	Anti-sense 5'-GGTGCAAGTGGACAGAGAGA-3'
Smad7	Sense 5'-GTTGGTGCACAAAGTGTTCC-3'
	Anti-sense 5'-GAAGCTAATCTGCACGGTGA-3'
